# Maternal Malaria, Birth Size and Blood Pressure in Nigerian Newborns: Insights into the Developmental Origins of Hypertension from the Ibadan Growth Cohort

**DOI:** 10.1371/journal.pone.0024548

**Published:** 2011-09-13

**Authors:** Omolola O. Ayoola, Isla Gemmell, Olayemi O. Omotade, Olusoji A. Adeyanju, J. Kennedy Cruickshank, Peter Ellis Clayton

**Affiliations:** 1 Endocrine and Cardiovascular Sciences Group, Manchester Academic Health Sciences Centre, University of Manchester, Manchester, United Kingdom; 2 College of Medicine, University of Ibadan, Ibadan, Nigeria; 3 Department of Obstetrics and Gynecology, Adeoyo Maternity Hospital, Ibadan, Nigeria; Walter & Eliza Hall Institute, Australia

## Abstract

**Background:**

Hypertension is an increasing health issue in sub-Saharan Africa where malaria remains common in pregnancy. We established a birth cohort in Nigeria to evaluate the early impact of maternal malaria on newborn blood pressure (BP).

**Methods:**

Anthropometric measurements, BP, blood films for malaria parasites and haematocrit were obtained in 436 mother-baby pairs. Women were grouped to distinguish between the *timing* of malaria parasitaemia as ‘No Malaria’, ‘Malaria during pregnancy only’ or ‘Malaria at delivery’, and parasite density as low (<1000 parasites/µl of blood) and high (≥1000/µl).

**Results:**

Prevalence of maternal malaria parasitaemia was 48%, associated with younger maternal age (p<0.001), being primigravid (p = 0.022), lower haematocrit (p = 0.028). High parasite density through pregnancy had the largest effect on mean birth indices so that weight, length, head and mid-upper arm circumferences were smaller by 300 g, 1.1 cm, 0.7 cm and 0.4 cm respectively compared with ‘No malaria’ (all p≤0.005). In babies of mothers who had ‘malaria at delivery’, their SBPs adjusted for other confounders were lower respectively by 4.3 and 5.7 mmHg/kg compared with ‘malaria during pregnancy only’ or ‘none’. In contrast the mean newborn systolic (SBP) and diastolic BPs (DBP) adjusted for birth weight were higher by 1.7 and 1.4 mmHg/kg respectively in babies whose mothers had high compared with low parasitaemia.

**Conclusions:**

As expected, prenatal malarial exposure had a significant impact on fetal growth rates. Malaria at delivery was associated with the lowest newborn BPs while malaria through pregnancy, which may attenuate growth of the vascular network, generated higher newborn BPs adjusted for size. These neonatal findings have potential implications for cardiovascular health in sub-Saharan Africa.

## Introduction

Hypertension is now a public health and economic problem in Sub-Saharan Africa with a prevalence up to 33% in urban areas in Nigeria [1], [2]. Its well-known complications and mortality occur at a younger age than in developed countries [3], [4]. In sub-Saharan Africa, malaria is hyperendemic, particularly in pregnancy with prevalence rates from 20 to 44% in Nigerian women [5]. Most cases of malaria in pregnancy are asymptomatic because of immunity acquired during previous exposures [5], [6]. However, asymptomatic infection still has significant consequences for maternal and infant health resulting in maternal anaemia and intrauterine growth retardation (IUGR), which causes 43% of preventable low birth weight babies (LBW, birth weight <2500 g), contributing to 75,000–200,000 infant deaths each year [7]–[9]. In Nigeria about 12–24% of newborns are LBW as a result of IUGR [10], [11].

In numerous global studies, adult risk of hypertension and other chronic disease is associated with LBW [12], [13]. In an emerging economy, LBW babies who show catch-up growth may be at particular risk of developing such disease in midlife [14]. These observations subsequently led to the ‘developmental origins’ hypothesis.

In newborns, their blood pressure (BP) correlates with birth weight [15]–[17]. There may be differences in the relationship between birth weight and BP in preterm babies small (SGA) and appropriate for gestational age (AGA) in the first week of life [18]. AGA babies showed the expected positive correlation between birth weight and BP while SGA babies did not [18]. There are limited data on newborn BP in African children, in particular exploring the relationships between their BP, birth size and exposure to malaria in utero.

Therefore, we tested the hypothesis that BP at birth would be higher and birth size would be smaller in babies whose mothers had malaria in pregnancy, defined by its timing during pregnancy and/or at delivery and the magnitude of parasite density. This in turn may affect the rate of BP rise through childhood and set the scene for hypertension in later life.

## Methods

### Ethics Statement

Ethical approval was obtained from the joint University of Ibadan / University College Hospital Ethics committee and the University of Manchester Ethics committee. The study protocol and the rationale for the study were explained carefully in appropriate language, most commonly Yoruba or English, with questions answered as needed and written informed consent was obtained from all participants. After the delivery of their babies, another written informed consent was also obtained for the participation of their babies in the study.

### Study Site

A semi-urban community, Yemetu-Adeoyo, Ibadan in Southwest Nigeria, where malaria transmission is perennial, was the site for the study. Families come from a range of socioeconomic backgrounds. The local community hospital, Adeoyo Maternity Hospital (AMH), the oldest maternity hospital in Nigeria dating from 1927, provides primary and secondary medical care with over 4000 deliveries each year.

### Participants

Healthy women aged 18–45 years presenting at AMH before 36 weeks gestation and residing within the catchment area of the study centre for at least 2 years were recruited. Singleton babies born at ≥37 weeks gestation were included.

At booking, women were tested for sexually transmitted infections and HIV and those positive were excluded and those with chronic diseases such as hypertension and diabetes. Preterm deliveries, babies with known syndromes, metabolic defects, congenital abnormalities or severe birth trauma were excluded.

Women were enrolled over one year to cover both wet and dry seasons.

In the year, 3496 women booked for ante-natal care, but many were not planning their delivery at AMH so 659 women were eligible, of which 624 were recruited but 161 still did not deliver at AMH; thus the final cohort included 463 mother-baby pairs. Of these, 27 were excluded due to 4 (0.9%) maternal deaths, 11 (2.4%) still births, 5 (1.1%) miscarriages and 7 (1.5%) neonatal deaths, leaving 436 pairs. There were no significant differences in the socio-demographic and clinical data of excluded women.

### Procedures: Time points and measurements

Standard operating procedures (SOPs) were developed. Informed consent, using forms translated into Yoruba, was taken at booking, then demographic, obstetric, family and health details, malarial history and use of antimalarial drugs was recorded. All women were issued with prescriptions for sulphadoxine-pyrimethamine (SP) for Intermittent Preventive Therapy (IPT) for malaria according to standard hospital practice.

#### Maternal Anthropometry

Standardized measures of anthropometry and BP were taken at every antenatal visit until delivery, weight to the nearest 0.1 kg (SECA scale), height on a stadiometer to the nearest 0.1 cm, both without shoes, according to the SOP and training video.

#### Blood Samples

At each visit throughout pregnancy, 2 ml of blood was obtained in EDTA tubes for haematocrit, leukocyte count and blood film for MP. For haematocrit, a capillary tube was filled to about 80% with blood, sealed with a flame and centrifuged for 5 minutes at 10,000 revolutions per minute. The haematocrit value was read with a heamatocrit reader. For the leucocyte count, anticoagulated blood at a one in 20 dilution in turk's solution was mixed for about 1 minute and placed in the counting chamber. The sample was left to settle, and the white cells were counted using a microscope (10× objective lens) in the four outer 1 mm^2^ areas. Total leucocyte number was calculated as 50× the number of cells counted.

Blood films were prepared, stained with 3% Giemsa at pH 7.2 and examined for malaria parasites (MP) under light microscopy. Thick smears were recorded as negative only after 200 high-power microscope fields had been scanned. In those with malaria, absolute parasite counts were determined as previously reported [19] by counting the number of parasites (np) among 200 leucocytes on the thick film as follows: Absolute parasite counts (per microlitre of blood) = (np / 200)×TLC where TLC = subject's total leucocyte count).

For quality control, 30% of negative samples and 40% of positive samples were re-examined by two different trained microscopists.

### Follow-up, Delivery and Recruitment of Babies

Women were followed up until delivery based on routine ante-natal practice, determined by gestational age. Repeat MP blood films were obtained every visit and at delivery when a film was also prepared from cord blood. The placenta was weighed, turned to the maternal surface, cotyledons exposed and 1 ml of blood obtained from the intervillous space for a placental malarial blood smear. Detailed delivery information was recorded.

#### Newborn Anthropometry and Skinfold Measures

Babies were weighed naked to the nearest 0.1 kg, length was measured from crown to heel on an infant stadiometer to the nearest 0.1 cm and occipito-frontal circumference (OFC) around the widest circumference of the head using a non-stretchable tape to the nearest 0.1 cm. Skinfold thicknesses (triceps, biceps, sub-scapular, and suprailiac) were measured using Holtain calipers on the left side to the nearest 0.1 mm. Measurements were obtained in duplicate or triplicate if disagreeing by >15%. All babies were examined within 72 hours of birth.

#### Maternal and Newborn BP

Maternal and newborn BPs were taken according to a standard protocol and SOP. Before the BP reading, the woman was comfortably seated and relaxed with the back and arm supported, the legs uncrossed, for at least 5 minutes and not moving or speaking. Her upper arm was supported at the level of the heart with no tight clothing constricting the arm. The measurement was carried out on the left arm with validated Datascope BP monitor using appropriate-sized cuffs (i.e. the bladder length and width of the cuff supplied were ≥80% and 40%, respectively, of the arm circumference).

Before performing the BP reading, the baby was comfortably lying on the mother's lap for at least 5 minutes, and many times they were asleep. The measurement was again done with the Datascope BP monitor, specifically validated for infants, using appropriate newborn cuffs on the left arm. Babies were measured within 72 hours of life. In both mother and child, measurements were repeated three times at least one minute apart and the mean of the last two readings used for analysis.

#### Validity of Anthropometric and BP Measurements

Three nurses trained in anthropometry and BP methods, based on the WHO manual (1995) and SOPs, carried out all measurements throughout the study on the same equipment. Inter-observer and within-observer error were minimized through 3-monthly refresher training sessions.

### Definitions

Anaemia was defined as packed cell volume (PCV)<30%.

Malaria was defined as asexual blood stages of Plasmodium falciparum in peripheral blood or placenta of the pregnant women or cord blood at delivery. All visits during pregnancy and at delivery were taken into account.

For this study, women were first grouped into 2 categories:

- ‘No Malaria’ - no parasites detected throughout pregnancy or at delivery (n = 225).
- ‘Malaria present’ - parasites present at least once during pregnancy and/or at delivery (n = 211).

Women with malaria were then stratified to distinguish between the *timing* of malaria through all visits in pregnancy and at delivery:

- ‘Malaria during pregnancy only’ - presence of malaria parasites at least once during pregnancy but not at delivery (n = 138).
- ‘Malaria at delivery’ - mothers with parasites present in the placenta and/or their peripheral blood sample at delivery and/or in the cord blood (n = 73).

To examine effects of parasite load during pregnancy and at delivery, parasite density was classified into low (<1000 parasites/µl), or high (≥1000/µl) based on the highest density recorded at any time point'.

### Statistical Analysis

Data were analysed using SPSS version 14 (SPSS Inc, Chicago, IL). Socioeconomic index scores were based on occupations and educational attainment of both parents on scales I to V, as previously [20]. Means of the four scores to the nearest whole number were assigned. Associations between categorical variables and malaria status were assessed using Chi-square tests and Odds Ratios. Differences in infant growth characteristics and blood pressure at birth were assessed using t-tests and ANOVA. Based on the results from the t-tests and ANOVA, multiple regression methods were used to determine which factors were independent predictors of birth weight, length, SBP and DBP. Malaria timing and parasite density were entered into the regression model as categorical variables with dummy variables. Based on the results from the univariate analyses, we have used parasite density in the regressions for birth weight and length and we have used malaria timing in the regressions for newborn SBP and DBP. Two-sided P values<0.05 were considered significant.

## Results

### Clinical characteristics of mothers and malaria status

All women recruited had at least 2 antenatal clinic attendances, 94% attended 3 times, 80% four times and 63% five times before delivery. The median (range) durations of the pregnancy at booking and at delivery were 28 (12–36) and 39 (37–42) weeks respectively with 28% of women being primigravida.

Parasitaemia was present at least once in pregnancy and/or delivery in 211 of the 436 recruited mothers (total 48%, with 30% having low parasitaemia and 18% high parasitaemia). Classified by *timing*, 138 (31%) had malaria parasitaemia at some time during pregnancy only and 73 (17%) at delivery.

56% of those with parasitaemia were primigravid, so malaria and first pregnancy were significantly associated (X^2^ = 5.276, p = 0.022) and associated with nearly a 3-fold increase in risk of having malaria (OR = 2.5, 95% CI, 1.5–4.2).

About half of the women reported the use of preventive measure such as chemoprophylaxis / insecticide spray or coil / bed nets / netted windows but these were not associated with protection from malaria (p>0.05). Social class and maternal malaria parasitaemia were also not associated (X^2^ = 1.557, p = 0.212).

Most women were asymptomatic. A complaint of fever in the week preceding recruitment and at every visit until delivery was reported in 9 women and fever recorded in only 6 women. Women with malaria had lower gravidity (p = 0.005), they were younger (27.7 vs 29.4 years, p = 0.001) and more likely to be anaemic than those without parasitaemia (*Table 1*). Malaria parasitaemia and anaemia was found in 19% of all women, but anaemia was present in 32%. Mean (SD) PCV was 32.2 (2.9)% in women without malaria, 31.9 (3.4)% in those with low parasitaemia and 30.7(3.6)% in those with high parasitaemia (p = 0.003).

**Table 1:** Clinical Characteristics of Mothers at recruitment by Malarial Status.

Parameter	Malaria Absent n = 225 (52%)		Malaria present n = 211 (48%)			
	Mean	SD	Mean	SD	t test	P value
Age (years)	29.4	4.8	27.7	5.1	3.50	**0.001**
Gravidity	1.9	1.6	1.5	1.3	2.80	**0.005**
GA at booking (weeks)	27.3	4.9	26.7	5.4	0.89	0.374
Body temperature (°C)	37.1	0.6	37.2	0.6	0.01	0.992
Weight ( kg)	64.5	11.5	63.9	10.8	0.53	0.596
Height (cm)	160.4	5.9	160.1	5.3	0.52	0.600
SBP (mmHg)	104.0	9.4	104.2	7.7	−0.20	0.840
DBP (mmHg)	60.9	7.2	61.0	6.7	−0.10	0.921
Packed cell volume (%)	32.2	2.9	31.5	3.5	2.21	**0.028**

GA – Gestational Age; SBP – Systolic blood pressure; DBP- Diastolic blood pressure.

There were no differences in other clinical characteristics such as pregnancy duration at booking, weight, height, body temperature, SBP and DBP in women with and those without malaria (*Table 1*).

### Clinical characteristics of babies and malarial status: parasite density compared with timing

#### Growth variables

Anthropometric variables and skinfolds of infants born to women with malaria parasitaemia were globally lighter than those of women without (*Table 2* *and* *3a*).

**Table 2:** Associations between maternal malarial status, newborn growth characteristics and newborn blood pressure at birth.

Parameter	Malaria Absentn = 225		Malaria Presentn = 211			
	Mean	SD	Mean	SD	t test	p value
Weight (kg)	2.97	0.4	2.85	0.4	2.72	**0.007**
Length (cm)	48.97	2.4	48.35	2.1	2.82	**0.005**
OFC (cm)	34.5	1.4	34.2	1.2	2.21	**0.028**
MUAC (cm)	9.95	0.8	9.77	0.9	2.11	**0.036**
Subscapular (cm)	4.28	0.9	4.14	0.8	1.67	0.097
Suprailiac (cm)	4.42	0.9	4.27	0.9	1.60	0.111
Triceps (cm)	4.19	0.8	4.08	0.9	1.28	0.200
Biceps (cm)	3.70	0.8	3.55	0.6	2.15	**0.032**
Subscap/Triceps Ratio	1.03	0.1	1.02	0.1	0.13	0.899
SBP (mmHg)	72.4	13.4	69.5	12.2	2.34	**0.020**
DBP (mmHg)	37.0	9.5	35.2	8.4	1.97	**0.049**

OFC – Occipito-frontal circumference; MUAC – Mid-upper arm circumference; Subscap – Subscapular skinfold thickness; SBP- Systolic blood pressure; DBP- Diastolic blood pressure.

**Table 3:** Associations between growth characteristics and blood pressure at birth defined by Parasite density and Timing of Malaria.

a. Parasite density#						b. TIMING of maternal malaria*				
Variable	Malaria	mean	Δ	95% CI of Difference	p-value+	Malaria	mean	Δ	95% CI of Difference	p-value+
**Weight kg**	None **n = 225**	3.0	0.3	0.1,0.4	**0.005**	No malaria **n = 225**	3.0	0.1	0.0, 0.2	**0.006**
	Low **n = 131**	3.0	0.3	0.1,0.4	**0.005**	In pregnancy **n = 138**	2.8	*		
	High **n = 80**	2.7	#			At delivery **n = 73**	2.9	0.1	−0.1, 0.2	0.425
**Length cm**	None	49.0	1.1	0.5,1.6	**0.001**	No malaria	49.0	0.7	0.2, 1.2	**0.006**
	Low	48.6	0.7	0.04,1.3	**0.037**	In pregnancy	48.3	*		
	High	47.9	#			At delivery	48.5	0.2	−0.5, 0.9	0.565
**OFC cm**	None	34.5	0.7	0.3,1.0	**<0.001**	No malaria	34.5	0.3	−0.0, 0.6	0.065
	Low	34.4	0.6	0.3,1.0	**0.001**	In pregnancy	34.2	*		
	High	33.8	#			At delivery	34.1	−0.1	−0.4, 0.3	0.792
**MUAC mm**	None	9.9	0.4	0.2,0.6	**0.001**	No malaria	10.0	0.3	0.0, 0.4	**0.024**
	Low	9.9	0.4	0.1,0.6	**0.006**	In pregnancy	9.7	*		
	High	9.5	#			At delivery	9.8	0.1	−0.1, 0.4	0.388
**Sub-scapular**	None	4.4	0.4	0.0,0.7	**0.026**	No malaria	4.3	0.2	0.0, 0.4	0.088
**mm**	Low	4.2	0.2	−0.1,0.6	0.199	In pregnancy	4.1	*		
	High	4.0	#			At delivery	4.2	0.1	−0.2, 0.4	0.467

+ **Analysis of Variance.**

#**Reference group = High parasitaemia.**

*Reference group = Malaria in pregnancy.

**There were no differences between Low parasitaemia and None.**

Δ – Difference; OFC – Occipito-frontal circumference; MUAC – Mid-upper arm circumference; Subs – Subscapular skinfold thickness;

Based on *parasite density*, birth weight, length, OFC, and MUAC of newborns born to women with high parasitaemia were smaller by 300 (95% CI 100–400)gm, 1.1 (0.5–1.6)cm, 0.7 (0.3–1)cm and 0.4 (0.2–0.6)cm respectively compared with those without parasitaemia (*Table 3a*). Skinfold thicknesses (biceps, triceps and subscapular) were also smaller than those whose mothers had low parasitaemia (*Table 3a* *and* *4a*).

**Table 4:** Associations between growth characteristics and blood pressure at birth defined by Parasite density and Timing of Malaria.

a. Parasite density#						b. TIMING of maternal malaria*				
Variable	Malaria	mean	Δ	95% CI of Difference	p-value+	Malaria	mean	Δ	95% CI of Difference	p-value+
**Triceps mm**	None	4.2	0.3	0.0,0.5	**0.031**	No malaria	4.2	0.1	0.0, 0.3	0.139
	Low	4.2	0.3	−0.2,0.5	0.074	In pregnancy	4.1	*		
	High	3.9	#			At delivery	4.1	0.0	−0.2, 0.3	0.453
**Biceps mm**	None	3.7	0.2	0.0,0.4	**0.029**	No malaria	3.7	0.2	0.0, 0.3	**0.024**
	Low	3.6	0.1	−0.1,0.3	0.365	In pregnancy	3.5	*		
	High	3.5	#			At delivery	3.6	0.1	−0.1, 0.3	0.446
**Suprailiac**	None	4.4	0.2	−0.0, 0.5	0.05	No malaria	4.4	−0.1	−0.0, 0.4	0.135
**mm**	Low	4.3	0.1	−0.1, 0.4	0.26	In pregnancy	4.3	*		
	High	4.2	#			At delivery	4.3	0.0	−0.3, 0.3	0.865
**Subs/Triceps**	None	1.0	0.0	−0.0, 0.0	0.717	No malaria	1.03	0.0	−0.0, 0.0	0.975
**Ratio**	Low	1.0	0.0	−0.0, 0.0	0.693	In pregnancy	1.03	*		
	High	1.0	#			At delivery	1.02	−0.01	−0.0, 0.0	0.857
**SBP mmHg**	None	72.4	3.0	−0.4,6.4	0.082	No malaria	72.4	1.4	−1.3, 4.3	0.292
	Low	69.5	0.1	−3.6,3.8	0.956	In pregnancy	71.0	*		
	High	69.4	#			At delivery	66.7	−4.3	−8.0, −0.6	**0.001**
**DBP mmHg**	None	37.0	1.0	−1.4,3.3	0.442	No malaria	37.0	1.1	−0.9, 3.0	0.294
	Low	34.7	−1.3	−3.9,1.3	0.329	In pregnancy	35.9	*		
	High	36.0	#			At delivery	33.9	−2.0	−4.7, 0.6	0.125

+ **Analysis of Variance.**

#**Reference group = High parasitaemia.**

*Reference group = Malaria in pregnancy.

**There were no differences between Low parasitaemia and None.**

Δ – Difference; Subs – Subscapular skinfold thickness; SBP- Systolic blood pressure; DBP- Diastolic blood pressure.

Analysis by *malaria timing* showed that parasitaemia at delivery had no additional impact on anthropometric variables compared to parasitaemia during pregnancy only (*Table 3b*).

Regression analyses testing effects on growth parameters showed that birth length, gestational age at birth and maternal weight were each independently associated with birth weight, while high parasite density was inversely related (*Table 5*). Only birth weight was independently related to birth length with no effect of malarial status (*Table 5*).

**Table 5:** Multiple regression analyses for determinants of birth size including maternal malaria parasite density.

Variable	Regression for Birth weight			Regression for Birth length			
	ß	95% CI	P-value		ß	95% CI	P-value
Length (cm)	0.10	0.08 to 0.11	**<0.001**	Weight (kg)	2.78	2.29 to 3.28	**<0.001**
GA at birth (weeks)	0.04	0.02 to 0.07	**0.003**		0.07	−0.08 to 0.22	0.37
No Malaria (Reference)+	-	-	-		-	-	-
Low Parasite density+	−0.02	−0.10 to 0.07	0.71		−0.26	−0.70 to 0.17	0.23
High Parasite density+	−0.10	−0.20 to −0.00	**0.045**		−0.28	−0.81 to 0.25	0.31
Maternal age (years)	0.00	−0.01 to 0.01	0.35		0.04	−0.01 to 0.09	0.15
Maternal weight (kg)	0.01	0.00 to 0.01	**<0.001**		0.01	−0.01 to 0.03	0. 45
Maternal height (cm)	−0.00	−0.01 to−0.00	0.22		0.03	−0.01 to 0.08	0.09
Maternal SBP (mmHg)	0.00	−0.01 to 0.01	0.88		−0.01	−0.05 to 0.02	0.49
Maternal DBP (mmHg)	−0.00	−0.01 to 0.01	0.58		−0.01	−0.05 to 0.03	0.73
Gravidity	−0.01	−0.05 to 0.02	0.49		−0.00	−0.20 to 0.20	0.99
Number of Antenatal Visits	0.01	−0.01 to 0.03	0.33		0.09	−0.04 to 0.21	0.17

GA – Gestational Age; SBP- Systolic blood pressure; DBP- Diastolic blood pressure.

+ **Parasite density: Coding 0 = No Malaria- Reference category,**

**1 = low parasite density; 2 = high parasite density.**

#### Impact of maternal malaria parasitaemia on Newborn BP

Babies whose mothers had no parasitaemia had higher mean SBP (p = 0.02) and DBP (p = 0.049) than those with parasitaemia (*Table 2*). This effect can be attributed to malaria timing: babies whose mothers had parasitaemia at delivery had SBP lower by 4.3 (0.6–8.0)mmHg/kg than those of women with parasitaemia in pregnancy only, and 5.7 (0.8–8.9)mmHg/kg lower than those without parasitaemia (*Table 4b*).

In contrast when evaluating the effect of parasite density through pregnancy (Table 4a), mean SBP and DBP were not different. However newborn BP is size dependent and when adjusted for weight SBP and DBP were higher by 1.7 (0.2, 3.3)mmHg/kg, p = 0.024 and 1.4 (0.4, 2.3)mmHg/kg, p = 0.006 respectively in babies whose mothers had high parasitaemia compared to those with low (*Table 6*).

**Table 6:** Association between maternal malaria parasite load and newborn SBP and DBP adjusted for birth weight and length.

Variable	Malaria Parasite density	mean	Difference	95% CI of difference	p-value+
SBP/weight	None	24.8	−0.9	−0.5, 2.3	0.185
	Low	24.0	−1.7	−0.2, −3.3	**0.024**
	High	25.7			
SBP/length	None	1.48	0.03	−0.11, 0.04	0.417
	Low	1.43	−0.02	−0.06, 0.10	0.663
	High	1.45			
DBP/weight	None	12.6	−0.7	−0.2,1.7	0.105
	Low	12.0	−1.4	−0.4, −2.3	**0.006**
	High	13.4			
DBP/length	None	0.75	0.0	−0.05, 0.05	0.958
	Low	0.72	−0.3	−0.02, 0.09	0.164
	High	0.75			

+ **Analysis of Variance.**

SBP- Systolic blood pressure; DBP- Diastolic blood pressure.

In regression analyses, newborn SBP was independently associated with birth weight and gestational age and inversely associated with birth length and maternal height. Malaria parasitaemia timing, specifically at delivery was also a determinant of infant SBP (*Table 7*). Maternal age, weight and DBP were not. Newborn DBP at birth was positively associated with birth weight, gestational age and inversely with parasitaemia at delivery (*Table 7*).

**Table 7:** Multiple regression analyses for determinants of newborn SBP and DBP at birth including maternal malaria timing.

*Variable*	*Regression for SBP*			*Regression for DBP*		
	ß	95% CI	P-value	ß	95% CI	P-value
Weight (kg)	8.35	4.36 to12.35	**<0.001**	3.07	0.26 to 5.88	**0.032**
Length (cm)	−1.2	−1.9 to −0.4	**0.003**	−0.2	−0. to 0.3	0.37
GA at birth (weeks)	1.39	0.4 to 2.4	**0.009**	0.90	0.17 to 1.62	**0.016**
No Malaria (Reference)*	-	-	-	-	-	-
Malaria in pregnancy*	−2.0	−5.0 to 1.0	0.19	−1.63	−3.7 to 0.5	0.13
Malaria at delivery ± pregnancy*	−6.6	−10.4 to 2.8	**0.001**	−4.06	−6.7 to −1.4	**0.003**
Maternal age (years)	0.32	−0.3 to 0.7	0.07	−0.04	−0.3 to 0.2	0.73
Maternal weight (kg)	−0.06	−0.2 to 0.09	0.41	−0.01	−0.1 to 0.1	0.82
Maternal height (cm)	−0.31	−0.6 to −0.05	**0.02**	−0.17	−0.4 to 0.01	0.06
Maternal SBP (mmHg)	0.19	−0.05 to 0.4	0.12	0.07	−0.1 to 0.2	0.40
Maternal DBP (mmHg)	−0.25	−0.5 to 0.04	0.09	0.00	−0.2 to 0.2	0.98
Gravidity	−0.66	−2.0 to0.7	0.33	−0.22	−1.2 to 0.7	0.64
Number of Antenatal Visits	−0.36	−1.2 to 0.5	0.40	0.06	−0.53 to 0.64	0.84

GA – Gestational Age; SBP- Systolic blood pressure; DBP- Diastolic blood pressure.

*Malarial timing: Coding 0 = No Malaria- Reference category 1 = malaria in pregnancy, 2 = malaria at delivery ± pregnancy.

## Discussion

### General features of malaria

This study illustrates the continuing impact of malaria in otherwise healthy pregnant Nigerian women, almost half of whom (48%) were affected. The rates confirm recent reports in pregnant Nigerian women [5], [21] and are also in agreement with findings from Malawi, Gabon and Ghana, but lower than in Western Kenya [22]–[25]. The latter areas have similar endemic rates to that in Nigeria. Most of our study cohort was asymptomatic, in line with previous findings that malaria in pregnancy in Africa rarely results in fever or any symptoms and therefore remains mostly undetected and untreated [8].

For those women reporting the use of preventive measures against malaria, including the 53% who said they used chemoprophylactic drugs, there was no difference in malaria parasitaemia frequency. Previous findings have shown that netted windows, insecticide sprays, mosquito repellent and insecticide-treated nets were effective in protecting against malaria [26], [27]. In Nigeria, there is low use of SP for IPT as recommended for prevention of malaria in pregnancy; this is due to cost, as was the case in this study. When used appropriately, IPT with SP is effective in preventing malaria in pregnancy [28]. In Mali, there was a reduction in the incidence of LBW among neonates born to women who used IPT with SP compared to other anti-malarial drugs [29].

In the suburban women in this study, malaria parasitaemia was not associated with social class, similar to a Kenyan report [24] but contrasting with those from Burkina Faso and India, with a higher incidence in low-income groups in rural areas [30], [31]. Primigravid women were more affected by malaria, as described [32], likely related to protective anti-adhesion antibodies against chondroitin sulphate A-binding parasites developing only over successive pregnancies [33].

In this study, diagnosis of malaria was based on microscopy of peripheral, placental and cord blood samples. Malaria in pregnancy still presents diagnostic challenges and has relied mainly on microscopy in studies in sub-Saharan Africa. Microscopy, though valuable, requires well-trained and skilled staff [34]. It can be used for speciation and quantification of parasites as done in this study, as well as assessing response to treatment. Histological examination, although reported to be more sensitive than microscopy on placental blood samples [35], was not available for this study. Placental blood, in addition to peripheral blood smears, were used to ensure that placental malarial infections were detected when peripheral parasitaemia may be negative [36]. Therefore we included all these sites in our definition of malaria in pregnancy and delivery.

### Birth outcomes

Babies of mothers with parasitaemia were globally smaller, most marked among babies of mothers with high parasite density during pregnancy. Similarly, mean birth weights in Burkina Faso, Tanzania, Mali and Pakistan for babies born to mothers with malaria in pregnancy were lower by 105 gm [37], 371 gm [38], 382 gm [32] and 461 gm [39] than those without malaria. Other anthropometry was not reported in these studies.

Birth weight is the single most important determinant of neonatal and infant survival and health, and malaria reduced all growth parameters, [40] probably related to chronic placental infection and insufficiency [40], [41].

### Effects on newborn BP

Some of the mechanisms linking small birth size and adult hypertension are poor maternal nutrition and maternal iron deficiency anaemia, which reduces vascular elasticity [42], [43]. In mothers with reduced skinfold thicknesses and lower haemoglobin concentrations, their children's SBP at the age of 10–12 years increased by 2.6 mmHg for each 1 g/dl decline in the mother's haemoglobin [42]. This confirms findings in experimental studies, where maternal iron restriction in the rat reduced birth weight and led to elevated BP at 40 days of age [44]. In this study anaemia at 32% is lower than previously reported in more rural Southern Nigeria but higher than rates from the nearby tertiary University College Hospital, Ibadan, with better obstetric facilities and managing patients in higher social classes [45], [46]. As elsewhere, anaemia is significantly associated with parasitaemia in primigravid women [5], [47]. These findings support the theory that maternal anaemia, an important consequence of malaria in pregnancy, could be linked to the genesis of raised BP in children. In contrast to other settings [48], maternal age was not associated with newborn BP, which is generally related to birth weight [49], [50].

In this cohort we found that babies whose mothers had malaria parasitaemia at delivery had lower SBP and DBP (Table 4). This observation could be accounted for in part by findings of lower mean BP in LBW babies and higher mean BP in those with higher birth weight [51], but this may also be related to the acute haemodynamic effects occurring in placental parasitaemia which may lead to lower newborn BP.

Overall, malarial load through pregnancy had the greatest impact on birth size (Table 3). Thus those babies who were exposed to high parasite loads through pregnancy were the smallest, and rather than having lower BP, both SBP and DBP corrected for weight were higher than in those exposed to a low parasite load (Table 6). This is in keeping with a developmental origins hypothesis linking placental insufficiency, IUGR and later hypertension. On-going follow-up will reveal whether small babies, having been exposed to intra-uterine malaria, who then show early catch-up growth, will have higher blood pressure. As far as we are aware, this is the first study to follow such a cohort, and the recognition of raised BP in early childhood could have important implications to health in Africa.

This leads to the question of how these two contrasting observations on BP related to timing and parasite density are mediated. Our hypothesis is that in these LBW babies, placental parasitaemia is associated with significant inflammation in the placental and infant arterial tree leading to more acute placental changes and significant infant vascular dilatation as an initial protective mechanism and hence lower BP at birth in those with parasitaemia at delivery. In contrast, parasitaemia *during* pregnancy leads to general growth restriction and smaller birth size and relatively higher BP for size. The more limited vascular tree of lighter infants may not be able to meet end-organ oxygen and nutritional demand without reactive peripheral vasoconstriction and higher BPs over time. Marginally but progressive higher BPs in infancy and early childhood may result, leading to a higher risk of hypertension in later life. Recurrent post-natal malaria will intermittently alter peripheral blood flow, but again at the expense of optimal supply to particular organs, hence restricting growth. The balance between the overall size of the fetus' vascular tree, how well particular organs grow during pregnancy, notably the renal glomeruli with their afferent and efferent arterioles, and continuing environmental hazards (e.g. infections, limited food supply and food quality) or opportunities (e.g. plentiful physical activity) will determine vascular performance, now measurable by pulse wave velocity [52].

### Conclusions

There is a high incidence of malaria and anaemia in this apparently healthy cohort of pregnant women, particularly the younger mothers and primigravids. Malaria in pregnancy adversely affects birth size, with high parasite density during pregnancy having the greatest impact on all growth parameters and being associated with higher BP corrected for weight. Follow-up studies to extend these observations into early childhood and to provide a better understanding of the influence of maternal malaria on BP in this cohort are underway.
